# Neuroprotective effect of peroxiredoxin 6 against hypoxia-induced retinal ganglion cell damage

**DOI:** 10.1186/1471-2202-11-125

**Published:** 2010-10-05

**Authors:** Rajkumar Tulsawani, Lorena S Kelly, Nigar Fatma, Bhavanaben Chhunchha, Eri Kubo, Anil Kumar, Dhirendra P Singh

**Affiliations:** 1 Department of Ophthalmology and Visual Sciences, University of Nebraska Medical Center, Omaha, NE, 68 198, USA; 2 Department of Neurosurgery, University of Nebraska Medical Center, Omaha, NE, 68198, USA; 3 Department of Ophthalmology, University of Fukui, Fukui, Japan; 4 Department of Pharmacology and Toxicology, University of Missouri- Kansas City, Kansas City, MO, 64108, USA

## Abstract

**Background:**

The ability to respond to changes in the extra-intracellular environment is prerequisite for cell survival. Cellular responses to the environment include elevating defense systems, such as the antioxidant defense system. Hypoxia-evoked reactive oxygen species (ROS)-driven oxidative stress is an underlying mechanism of retinal ganglion cell (RGC) death that leads to blinding disorders. The protein peroxiredoxin 6 (PRDX6) plays a pleiotropic role in negatively regulating death signaling in response to stressors, and thereby stabilizes cellular homeostasis.

**Results:**

We have shown that RGCs exposed to hypoxia (1%) or hypoxia mimetic cobalt chloride display reduced expression of PRDX6 with higher ROS expression and activation of NF-κB. These cells undergo apoptosis, while cells with over-expression of PRDX6 demonstrate resistance against hypoxia-driven RGC death. The RGCs exposed to hypoxia either with 1% oxygen or cobalt chloride (0-400 μM), revealed ~30%-70% apoptotic cell death after 48 and 72 h of exposure. Western analysis and real-time PCR showed elevated expression of PRDX6 during hypoxia at 24 h, while PRDX6 protein and mRNA expression declined from 48 h onwards following hypoxia exposure. Concomitant with this, RGCs showed increased ROS expression and activation of NF-κB with IkB phosphorylation/degradation, as examined with H2DCF-DA and transactivation assays. These hypoxia-induced adverse reactions could be reversed by over-expression of PRDX6.

**Conclusion:**

Because an abundance of PRDX6 in cells was able to attenuate hypoxia-induced RGC death, the protein could possibly be developed as a novel therapeutic agent acting to postpone RGC injury and delay the progression of glaucoma and other disorders caused by the increased-ROS-generated death signaling related to hypoxia.

## Background

Uncontrolled rises in intracellular reactive oxygen species (ROS) are triggered by downregulation of expression and activity of protective molecules in response to changes in the extracellular environment. Such changes often include hypoxia, the scarcity of oxygen can lead to cell injury and death by apoptosis. Recent evidence has shown an increase of intracellular ROS expression in cells during hypoxia, with the source of the increase being the mitochondria [1,2]. Mammalian cells respond to fluctuations in their micro environmental oxygen by regulating defense genes such as stress response genes, heat shock factor, NF-κB, and HIFα-1. These factors play a decisive role in the fate of cells by activating protective molecules such as PRDX6. However, a scarcity of oxygen to cells also results in functional or adaptive responses [3-6]. Conversely, prolonged hypoxia can induce genes involved in cell death [7,8]. The increased levels of ROS during hypoxia and ROS-driven-oxidative stress induce deleterious effects by activating/deactivating genes and deregulating normal survival signaling [9,10]. This process results in pathophysiology of cells and tissues, such as stroke, cardiovascular disease, tumorigenesis, and development of various blinding eye conditions [11,12]. The death of retinal ganglion cells (RGCs) is a major blinding event, and RGC death has been reported to occur during retinal hypoxia/ischemia [13,14]. Retinal cells that are highly active require a regular supply of oxygen [11,15]. Any interruption in oxygen supply due to an abnormality in circulation such as retinal artery occlusion or retinal vein thrombosis or atherosclerosis results in retinal hypoxia/ischemia. An extended period of hypoxia leads to the development of complications such as glaucoma, optic neuropathies, diabetic retinopathies, and retinal vein occlusions [16-19]. It has been found that the inner retina is more susceptible to hypoxia, in contrast to outer one [20]. To cope with oxidation-induced adverse effect one natural protective characteristic of eye is that intra-ocular O_2 _tensions are low however, many other cellular defense systems are evolved such as glycolysis, angiogenesis, vasodilation, and erythropoiesis in response to hypoxia [21], but these protective phenomenon are momentary [22], following which cell death and tissue damage occur [11]. Hypoxia-induced generation of ROS results in imbalance of the cellular oxidant-antioxidant status that leads to failure of cellular homeostasis. ROS-driven oxidative stress is a known cause of lipid peroxidation, protein oxidation, and DNA oxidation, which contribute to neurodegeneration [21,23,24]. Oxidative stress also has been reported to be cytotoxic to RGCs [10,25], causing necrotic or apoptotic death [10,22,26,27]. In addition, the generation of ROS is associated with activation or deactivation of several survival factors [28]. NF-κB is a transcription factor which is activated by various stimuli including oxidative stress. NF-κB plays multiple roles in cell survival, proliferation, and differentiation and also in cell death as a pro- or anti-apoptotic transcription factor, depending on cell type or the nature of injury [29,30]. A wealth of information documents that RelA containing NF-κB complex has an antiapoptotic effect [31]. In glaucoma, NF-κB is highly activated in RGCs and has been suggested to be proapoptotic and implicated in retinal neuronal cell death [32,33]. Initiation of apoptotic response to a variety of stress signals via NF-κB requires its translocation into nucleus from the cytoplasm. Under normal circumstances, in cell cytoplasm an association between IkB, an inhibitor protein, and NF-κB dimers renders NF-κB inactive. IkB is a member of a family of regulator proteins, viz. IkB-α, IkB-β, and Bcl3. However, in response to stress signals, IkB undergoes phosphorylation, which releases its inhibition of NF-κB. NF-κB translocates to the nucleus and binds to DNA [10,34].

Moreover, the over-expression of intracellular ROS caused by extracellular stressors is controlled by antioxidant defenses such as catalase, SOD, glutathione peroxidase, and, most importantly, the newly discovered peroxiredoxins [35-39]. The peroxiredoxin (Prdx) family includes six known members (Prdx 1-6). Of particular interest is PRDX6 cloned by our group from human lens epithelial cells cDNA library [40]. This agent has GSH peroxidase as well as acidic Ca^2+^-independent phospholipase A2 activities [37,39-44]. Recently, our group has shown that PRDX6 has protective potential in saving RGCs against glutamate and TNF-α induced cytotoxicity. It acts by limiting ROS and maintaining calcium homeostasis [10,37,42,43,45-48]. The unique ability to regulate signaling and to maintain phospholipid turnover distinguishes PRDX6 from the other five peroxiredoxins (Prdx1 to 5). This molecule is widely expressed, occurring in high levels in the liver, lung, eye lens, and keratinocytes [37,40,48-50] including RGCs [9], and its reduced expression can lead to cell death and tissue degeneration [47,48,51]. Recently, PRDX6 has been implicated in maintaining blood vessel integrity in wounded skin [52,53] and in development and progression of several diseases, including oxidative-induced cataractogenesis [43,54], psoriasis [55,56], atherosclerosis [51], and parkinsonian dementia [57]. Thus, accumulating evidence indicates that underexpression of PRDX6 contributes to pathophysiology of cells and tissues, and this involves an increase in ROS levels. The increase leads to declines in a number of physiological functions because of overmodulation of ROS-mediated gene expression and activation of factors, including NF-κB. Stimulation of such factors in RGC has been implicated as a cause of the initiation of death signaling [58,59]. However, given the role of PRDX6 in maintaining cellular homeostasis by blocking death signaling and thereby regulating ROS expression [9,10,37], we think that a supply of PRDX6 will attenuate the hypoxia-evoked ROS-induced deleterious signaling in RGCs. In the study described here, we used RGCs exposed to 1% O_2 _and/or CoCl_2_, a hypoxia mimetic, as a model system to explore the underlying event(s) of hypoxia-induced RGC death. We have shown that RGCs facing hypoxia for longer periods display elevated expression of ROS and reduced levels of PRDX6, and that RGCs over-expressing PRDX6 gain resistance against hypoxia-evoked generation of ROS and ROS-induced cellular insults, by negatively regulating death signaling.

In this study, we investigated whether chronic hypoxia evoked the production of ROS in RGCs, and whether over-expression of ROS initiated NF-κB-mediated death signaling by phosphorylation/degradation of IkB, resulting in RGC death. In addition, by using transfection and transactivation assays, we showed that ROS-mediated suppression of Prdx6 mRNA and protein expression in RGCs bearing higher levels of ROS could be attenuated by the over-expression of PRDX6. Collectively, these findings provide a foundation for rational use of antioxidant-based therapeutics for treating or preventing/delaying RGC death from ROS driven oxidative stress under hypoxic conditions.

## Methods

### Culture of the retinal ganglion cell

RGC-5 (a kind gift from Neeraj Agarwal, University of North Texas Health Science, Fort Worth, TX, USA) were maintained in Dulbecco's modified Eagle's medium (DMEM) containing 10% fetal bovine serum (FBS), 100 U/ml penicillin and 100 μl/ml streptomycin at 37°C with 5% CO_2_. The cells reaching confluency were trypsinized and subcultured using 1:20 split. RGCs of 5 to 7 passages were used to carry out the experiments.

### Generation of Hypoxic conditions

Cells were either exposed to 1% oxygen using hypoxic chamber or they were treated with cobalt chloride, a hypoxia mimic [60,61] at various concentrations and for various time intervals.

### Cell survival assays: MTS and TUNEL assays

Cells (1 × 10^4^) were grown in 48 well plates and treated with 50, 100, 150, 200 or 400 μM of CoCl_2 _for 24, 48 or 72 h. After treatment period, a colorimetric MTS assay (Promega) was performed as described earlier [37]. This assay of cellular proliferation uses 3-(4,5-dimethylthiazol-2-yl)-5-(3-carboxymethoxyphenyl)-2 to 4-sulfophenyl)-2H-tetrazo-lium salt (MTS; Promega, Madison, MI). Upon being added to medium containing viable cells, MTS is reduced to a water-soluble formazan salt. The A_490 nm _value was measured after cobalt chloride treatment at specified duration with an ELISA reader. The values are represented as a percent change to matched controls within cell types.

A TUNEL assay was employed to assess and validate apoptotic cell death. TUNEL staining was performed using an in situ cell death detection kit, Fluorescein (Roche Diagnostics GmbH, Germany), following the company's protocol. Briefly, cells were grown in 4 chambered slide, washed with PBS and fixed in freshly prepared 4% paraformaldehyde in PBS (pH 7.4), followed by incubation in permeabilization solution (0.1% Triton X-100, 0.1% sodium citrate) for 2 min on ice. Cells were rinsed twice with PBS, and incubated in a TUNEL reaction mixture for 60 min at 37°C in the dark. Cells were rinsed three times with PBS. After mounting, samples were microphotographed using a micro-scope (Nikon, ECLIPSE TE 300), and analyzed. To determine the total dead cells, other than only the apoptotic cells, RGCs were stained with trypan blue solution (0.4%), where non viable cells are stained with trypan blue which is normally excluded by the live cells.

### Construction of Prdx6 Promoter-Chloramphenicol Acetyl-transferase (CAT) Reporter Vector

The 5'-flanking region from *-*1139 to +109 bp was isolated from mouse genomic DNA and sequenced [10]. A construct of -1139 bp was prepared by ligating it to basic pCAT vector (Promega) using the SacI and XhoI sites. Similarly, construct of deletion mutants of different sizes (- 839 to + 109 bp, construct B; -430 to + 109 bp, construct C) of the Prdx6 promoter with appropriate sense primers bearing SacI and reverse primer with XhoI were made [10]. The plasmid was amplified and used for the CAT assay. Primers were as follows: Construct A_for_, 5'-CTGAGAGCTCCTGCCATGTTC-3'; Construct B_for_, 5' CTTCCTCTGGAGCTCAGAATTTAC-3'; Construct C_for_, 5'-CACAG-AGCTCGTTCTTGCCACATC-3'; Constructs A, B, and C_rev_, 5'-CAGGAACTCGAGGAAGCGGAT-3'. We used construct B in the present study.

### Assay for intracellular redox state

Intracellular redox state levels were measured using the fluorescent dye, H2-DCFH-DA as described earlier [37,43]. Briefly, cells were washed once with HBSS and incubated in the same buffer containing 5-10 μg of DCFH-DA for 30 min at 37°C. Intracellular fluorescence was detected with Ex485/Em530 using Spectra Max Gemini EM (Molecular Devices, CA).

### Western analysis

Nuclear, cytoplasmic extracts and total Cell lysates were prepared as described previously [10]. Equal amounts of protein samples were loaded onto a 10% SDS gel, blotted onto PVDF membrane, and immune-stained with primary antibodies; PRDX6 monoclonal antibody (1:1000) (Lab Frontier, S. Korea), NF- κB (p65) (Santa Cruz Biotech) and β-actin antibody (Sigma) (1:2000). The membranes were further incubated with horseradish peroxidase-conjugated secondary antibodies (1:1500 dilution) following washing. Specific protein bands were visualized by incubating the membrane with luminol reagent (Santa Cruz Biotechnology) and exposing to film (X-OMAT; Eastman Kodak).

### Real-time PCR

To monitor the levels of Prdx6, NF- κB (p65), and β-actin mRNA in RGCs, total RNA was isolated using the single-step guanidine thiocyanate/phenol/chloroform extraction method (Trizol Reagent; Invitrogen) and converted to cDNA using Superscript II RNAase H-Reverse Transcriptase. Quantitative real-time PCR was performed with SYBR Green Master Mix (Roche Diagnostic Corporation, Indianapolis, IN) in a Roche^® ^LC480 Sequence detector system (Roche Diagnostic Corporation). We used primers specific for Prdx6 (forward 5'-TTGATGATAAGGGCAGGGAC-3' and reverse, 5'-CTACCATCACGCTCTCTCCC-3'), NF-κB (forward 5'-TTTCCCCTCATCTTTCCCTC-3' and reverse 5'-TGTGCTTCTCTCCCCAGG-3') and β-actin (forward 5'-CGTGGGCCGCCCTAGGCACCA-3' and reverse 5'-TTGGCCTTAGGGTTCAGGGGGG-3'). The primers were synthesized at University of Nebraska Medical Center, DNA Facility, Omaha, NE, USA. The comparative Ct method was used to calculate relative fold expression levels using the e-Roche software. The Cts of target genes were normalized to the levels of β-actin as an endogenous control in each group.

### Expression and purification of GFP-PRDX6 fusion protein

A full length cDNA of Prdx6 was isolated from human lens epithelial cell cDNA library using sense (5'-ATGCCCGGAGGTCTGCTTCTCGGGG-3') and antisense (3'-AATTGGCAG CTGACATCCTCTGGCTC-3') primers, and the resultant amplified product was cloned in pcDNA3.1/NT-GFP-TOPO vector procured from Invitrogen [40]. This construct was used for over-expressing PRDX6 in RGCs. Cells transfected with empty GFP vector served as control.

### Statistical method

Data are presented as Mean ± S.D. of the indicated number of experiments. Data were analyzed by Student's t-test when appropriate. A p value of < 0.05 was defined as indicating a statistically significant difference.

## Results

### Hypoxia induced RGC death with apoptosis, and these cells harbored elevated ROS, reduced PRDX6, and increased NF-κB expression

Recently reports have documented that hypoxia-induced elevation in intracellular ROS is a cause of pathophysiology in cells and tissues [62]. However, hypoxia exerts both proapoptotic and antiapoptotic biphasic effects that seem to be associated with cell types and conditions surrounding the cells. In the present study, we initially used the hypoxia-mimetic agent cobalt chloride (CoCl_2_) to expose RGCs to hypoxic stress. We examined whether RGCs exposed to such stress showed reduced survival and died with apoptosis and displayed higher ROS levels. Results from cells exposed to cobalt chloride were compared with those from RGCs exposed to 1% O_2_.

**Figure 1:**
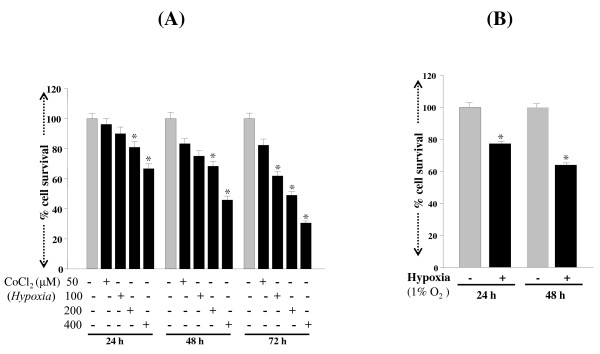
**MTS assay showing effect of hypoxia on the viability of RGCs**. Cells (1 × 10^4^) were cultured in 48-well plate containing DMEM supplemented with 10% FBS. 24 h later, cells were washed and treated with variable concentrations of cobalt chloride (A; black bars 50, 100, 200 or 400 μM) or exposed to 1% O_2 _(B; black bar) for a period of 24, 48 or 72 h. Results are means ± SE of three individual experiments. *p < 0.05.

**Figure 2:**
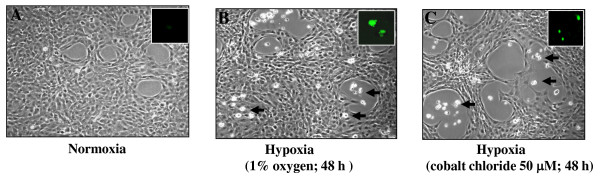
**Photomicrograph of RGCs without (A) or with CoCl_2 _(B) or exposed to 1% O_2 _(C)**. Arrow heads denote dead cells. TUNEL staining was performed as described in 'Methods' section to determine apoptotic RGC death following treatment. *Insets*: Photomicrograph of TUNEL-positive cells (green fluorescent) treated without (A) or with (B) or exposed to 1% O_2 _(C).

Cells exposed to cobalt chloride showed decrease in cell survival, with levels depending on concentration and exposure time (Figure 1A; black bars). Exposure of RGCs to 1% oxygen for 24 h or 48 h resulted in cell death in a time-dependent manner (Figure 1B; black bars). With both types of exposure, the cellular effects produced by hypoxia were associated with concentration as well as the duration of exposure. These cells were photomicrographed and recorded (Figures 2A-C. arrow; dead cells). The mode of RGC death was apoptosis following treatment with CoCl_2 _(Figure 2B) or 1% O_2 _exposure (Figure 2C) after 48 h when compared to unexposed cells (Figure 2A), as shown by TUNEL assay (Figure 2* insets*).

**Figure 3:**
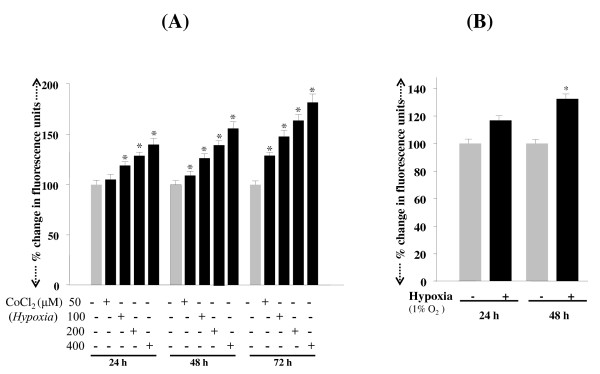
**Histogram showing intracellular ROS expression in RGCs following CoCl_2 _treatment or 1% O_2 _exposure**. Cells were treated with different concentrations of cobalt chloride (50, 100, 200 or 400 μM) (A) or exposed to1% oxygen (B) for a period of 24, 48 or 72 h. ROS were measured with H2-DCFH-DA dye. Results are means ± SE of 3 experiments. *p < 0.05

Next, to determine whether RGCs exposed to either CoCl_2 _or 1% O_2 _bore higher levels of ROS, we monitored the levels with the fluorescent dye, H2-DCFH-DA. Consistent with earlier reports, an increase in ROS levels was observed in cells exposed to hypoxic stress, and the levels of ROS were increased with increased time of exposure (Figures 3A and 3B; black bars).

**Figure 4:**
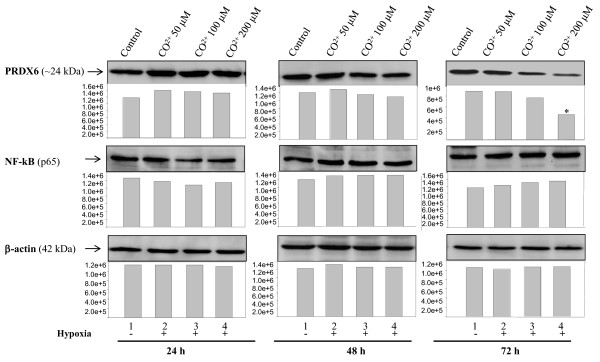
**Western analysis showing modulated expression of PRDX6 and NF-κB protein in RGCs treated with CoCl_2_**. Cells (4 × 10^5^) were cultured in 100 mm culture plates, and after 24 h these cells were treated with different concentrations of CoCl_2 _(50, 100, 200 μM) for a period of 24, 48 or 72 h. After treatment, cell extracts were prepared for Western analysis. A significant reduction of PRDX6 protein expression was observed following treatment with 100 or 200 μM of CoCl_2 _at 72 h (upper panel, PRDX6; 72 h; lanes 2, 3 and 4, 48 h; lanes 3 and 4). In contrast, an increase in NF-κB protein level was observed (middle panel) while no change was detected in the expression of β-actin level (lower panel), suggesting hypoxia specifically reduced the expression of PRDX6. Histogram shows relative density (Pixels) of protein bands. *p < 0.05.

**Figure 5:**
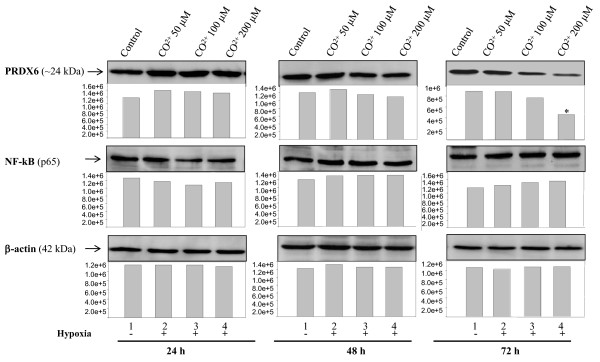
**Effect of Hypoxic stress on regulation of PRDX6 and NF-κB proteins in RGCs exposed to 1% O_2_**. Cells (4 × 10^5^) were cultured in 100 mm culture plates, and after 24 h these cells were exposed to 1% O_2 _for a period of 24 and 48 h. After treatment, cell extracts were prepared for Western analysis. A significant increase in PRDX6 protein expression was observed after 24 h and reduced after 48 h of 1% O_2 _exposure (lane 2). In contrast, an increase in NF-κB protein level was observed (middle panel) while no change was detected in the expression of β-actin level (lower panel), suggesting hypoxia specifically reduced the expression of PRDX6. Histogram shows relative density (Pixels) of protein bands. *p < 0.05.

Because reduced expression of intracellular PRDX6 is causally related to increase expression of ROS, and NF-κB is a regulator of PRDX6 in cells in the redox state [9,10,43,45], we next examined whether levels of these two molecules were altered in cells under conditions of hypoxia. We conducted Western analysis with PRDX6-specific antibody, and the same blotted membrane was reprobed with NF-κB antibody following restriping. As expected, reduced expression of PRDX6 (Figure 4, PRDX6, 48 h or 72 h, lanes 2-4; Figure 5, PRDX6, 48 h, lane 2) and elevated expression of NF-κB (Figure 4, NF-κB, 24, 48 or 72 h, lanes 2-4; Figure 5, NF-κB, 24 or 48 h, lanes 2 and 4) were observed in cells exposed to hypoxia (48 h onwards) (Because the higher dose of cobalt chloride [400 μM] caused ~ 70% of the RGC death, that dose was excluded). Levels of PRDX6 were found to be increased when analyzed after 24 h of hypoxia exposure generated by cobalt chloride (50, 100 or 200 μM) or 1% oxygen (Figure 4, PRDX6, 24 h, lanes 2-4; Figure 5, PRDX6, 24 h, lane 2). The data illustrate the role of PRDX6 in RGC survival under hypoxia exposure.

### Modulation of PRDX6 and NF-κB in RGCs during hypoxia was due to repression of their transcription

**Figure 6:**
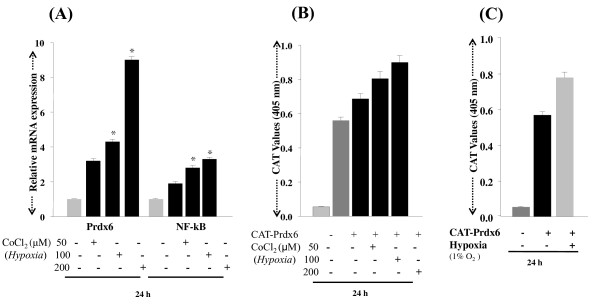
**Quantitative PCR showing differential expression of Prdx6 and NF-κB mRNA in RGCs with or without treatment with CoCl_2 _or exposed to 1% O_2_**. Total RNA was isolated and transcribed into cDNA. PCR was performed using specific primers as described in 'Methods' section. mRNA expression of each Prdx6 and NF-κB was adjusted to the mRNA copies of β-actin. Results indicate that mRNA expression level of Prdx6 was significantly increased after 24 h of exposure to different concentrations of CoCl_2 _(A; 50, 100 or 200 μM) with an increase of NF-κB mRNA. (B) CAT assay showing promoter activity of Prdx6 following hypoxia. Cells were transiently transfected with Prdx6-CAT (B & C). After 24 h of transfection, cells were either treated with cobalt chloride (50, 100 or 200 μM) (B) or exposed to 1% O_2 _(C; black bar). Transactivation assay was performed after 72 h of transfection. Results indicate that Prdx6 transcription (B and C) was up-regulated following cobalt chloride or 1% O_2 _exposure, respectively. *p < 0.05.

Although it was confirmed at the protein level that the expression of PRDX6 and NF-κB was modulated under hypoxia, it was not clear whether the changes in expression were due to modulation in the translation or in the transcription of these molecules. To clarify the regulation of PRDX6 and/or NF-κB by hypoxic stress at the transcriptional level, the cells were exposed to cobalt chloride (50, 100 or 200 μM) or to 1% O_2 _as described above for 24 h, and the mRNA from these cells was used to conduct real time PCR. Results demonstrated that RGCs exposed to hypoxia had an abundance of Prdx6 mRNA, but levels of NF-κB mRNA were not found to be dramatically high when compared to the controls (Figure 6A; black bars). To validate further activation of PRDX6 during hypoxia, we performed transactivation assays as described in Methods. RGCs were transfected with Prdx6 promoter linked to CAT construct B [10], and were exposed to hypoxia either by treating the cells with cobalt chloride (50, 100 or 200 μM) or by exposing to 1% oxygen. After exposure to various concentrations of cobalt chloride (Figure 6B; black bars) or 1% oxygen (Figure 6C; black bar) a pronounced activation in Prdx6 promoter activity could be observed, suggesting that Prdx6 is transcriptionally regulated in RGCs under hypoxia. This finding indicates that Prdx6 is upregulated to counteract hypoxia-induced cellular damage mediated via NF-κB, which plays an apoptotic role in RGC death.

### Hypoxia induced activation of NF-κB and its translocation into nucleus was associated with IkB phosphorylation/degradation

**Figure 7:**
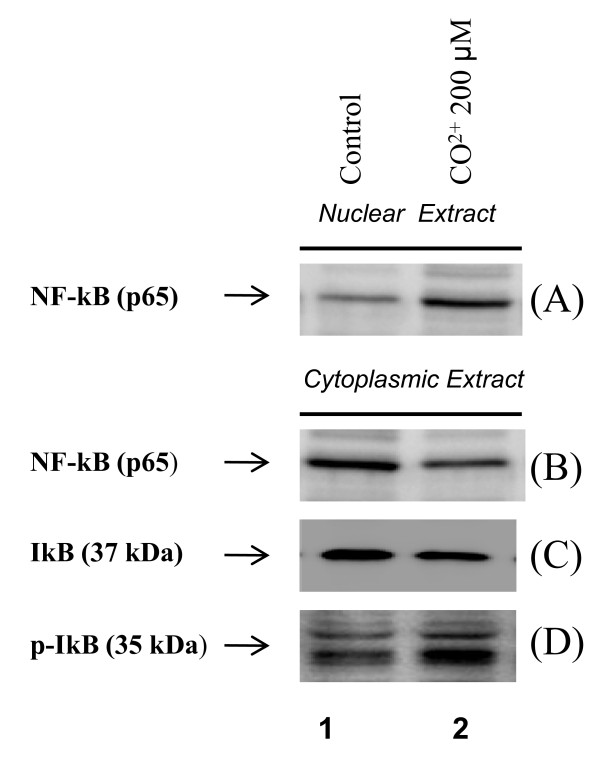
**Western analysis showing NF-κB translocation in nucleus from cytoplasm and phosphorylation of IkBα in RGCs facing hypoxic stress**. Cell (4 × 10^5^) were cultured in 10 mm culture plates and were treated with 200 μM of CoCl_2 _for 2 h. After treatment, cytoplasmic and nuclear extracts were prepared for western analysis. Cells treated with CoCl_2 _showed increased expression of NF-κB in nucleus (A, NF-κB, lane 2) with concomitant decrease in cytoplasmic expression (B, NF-κB, lane 2). These cells showed reduced expression of IkB (C; lane 2) and enhanced expression of its phosphorylated form pIkB in cytoplasm (D; lane 2).

To examine the dynamics of NF-κB and IkB interactions under hypoxia, cells were exposed to 200 μM of cobalt chloride and subcellular fractions were assayed by Western blot NF-κB (p65) nuclear translocation (Figure 7A) and IkB phosphorylation/degradation (Figure 7C). Under normal circumstances, cytoplasmic NF-κB is inactive through interactions with an inhibitor protein IkB. The phosphorylation of IkB unmasks the nuclear translocation signal on the NF-κB. In the present study, cells treated with cobalt chloride showed decreased expression of IkB (Figure 7C; IkB; lane 2), with an increase in phospho-IkB levels (Figure 7D; pIkB, lane 2). The results revealed that kinetics of the increase in p65 in nucleus mirrors the kinetics for phosphorylation and degradation of IkB in cytoplasmic fraction. Further activation of NF-κB was confirmed by transactivation assay; RGCs transfected with pLTR-CAT construct, which consists of two NF-κB sites [43], were subjected to hypoxic stress (1% O_2_) for 24 h. CAT activity assessed with CAT-ELISA as described in Methods revealed activation of NF-κB (Figure 9C, dark gray bar). Collectively, the findings demonstrate activation of NF-κB by hypoxia.

### PRDX6 over-expression attenuated hypoxia-induced RGC death, by reducing ROS production and optimizing NF-κB activation

**Figure 8:**
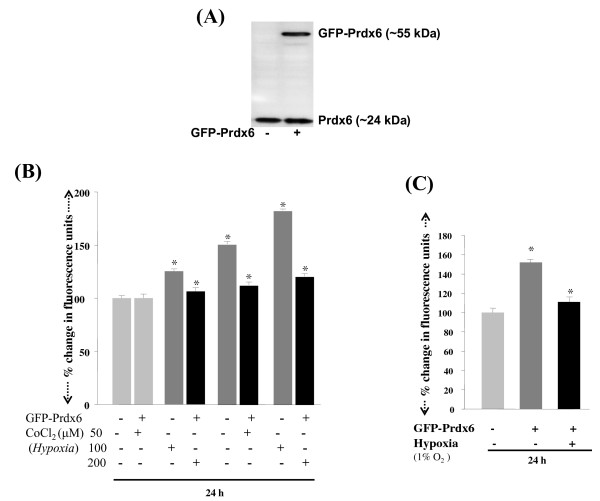
**Effect of over-expression of GFP-PRDX6 on ROS levels in RGCs following hypoxic stress**. Cellular extract was prepared and resolved on SDS-PAGE, and Western analysis was performed using antibody specific to PRDX6, to assess both exogenous and endogenous expression of PRDX6. ~55 kDa protein band was detected as recombinant GFP-PRDX6 protein (A, right lane). Histogram showing ROS levels in RGCs after exposure to hypoxia (B&C). Cells over-expressed with GFP-PRDX6 prevented excessive generation of ROS against CoCl_2 _(50, 100 or 200 μM). Cells were transiently transfected with either GFP vector or GFP-PRDX6. After 24 h cells treated with CoCl_2 _(50, 100 or 200 μM).

**Figure 9:**
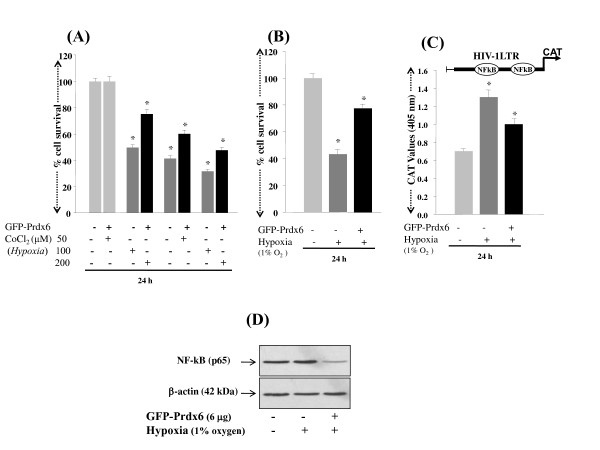
**PRDX6 regulation of NF-kB expression and cellular survival during hypoxia (A)**. MTS assay showing protective effect of over-expression of PRDX6 on RGCs survival. Cells were transiently transfected with either GFP vector or GFP-PRDX6. After 24 h cells were either treated with CoCl_2 _(50, 100 or 200 μM) or exposed to 1% O_2. _Results indicate that GFP-PRDX6 over-expression significantly reduced death of RGCs exposed to CoCl_2 _(A; 50, 100 or 200 μM) or 1% O_2 _(B). To monitor the activation of NF-κB, cells were transfected with GFP vector or GFP-PRDX6. These cells were co-transfected with HIV-1LTR-CAT construct (C) having NF-κB sites [37] and were exposed to 1% O_2_. Results showed the repression of NF-κB-dependent transcriptional activation of HIV-1LTR in cells over-expressing PRDX6 (C; black bar). In parallel experiments, cell extracts was prepared and Western analysis performed to confirm whether GFP-PRDX6 over-expression reduced the expression of NF-κB (D). Upper panel showing decreased expression of NF-κB in nuclear extract of RGCs over-expressing PRDX6.

PRDX6 exerts its protective function by regulating ROS expression and blocking cell death signaling. To determine the efficacy of PRDX6 in abolishing hypoxia-evoked ROS-driven damage to and death of RGCs, we transfected RGCs with pGFP-PRDX6 (2, 4 and 6 μg) as described in Experimental Procedures, and transfection efficiency was equalized with OD obtained from GFP fluorescence at Ex485/Em530. We also confirmed over-expression of pGFP-PRDX6 using Western analysis (Figure 8A) As expected, RGCs over-expressing PRDX6 showed reduced levels of ROS when they were exposed to either cobalt chloride (Figure 8B; 50, 100 or 200 μM; black bars) or 1% oxygen (Figure 8C; black bars). Cell death was reduced by ~20% to 30% as observed in these cells treated with various concentrations of the agents compared to controls transfected with empty GFP vector (Figure 9A; black bars). Similar results were obtained when PRDX6 over-expressed cells were exposed to 1% oxygen (Figure 9B; black bars). To test whether PRDX6 administration attenuated NF-κB activation in RGCs following hypoxia treatment, we did transactivation assay in RGCs using pLTR-CAT construct (Figure 9C). Results showed an increase in the promoter activity following hypoxia (1% oxygen) and the increase could be inhibited by a supply of PRDX6 (Figure 9C; black bar). Western analysis further confirmed that extrinsic supply of PRDX6 reduced expression of NF-κB in RGCs exposed to hypoxia (Figure 9D; NF-κB; lane 3). Taken together, the results demonstrated that PRDX6 protects against hypoxia-evoked oxidative stress mediated cell death by restoring survival signaling, at least in RGCs, and it does so by optimizing ROS expression and NF-κB activation and expression.

## Discussion

Changes in oxidant and antioxidant balance may alter cellular homeostasis; cells may go through survival or death pathways, depending upon expression of ROS and antioxidant defense capacity. Eye tissues are constantly exposed to external and internal environmental stresses such as sunlight, chemical irritations, and hypoxia. These stressors may lead to blinding disorders of the eye by inducing abnormalities in the homeostatic system of cells. Interruption of normal blood flow to an organ causes ischemia-hypoxia, which can result in tissue injury in many organs, including the heart, liver, lungs, and retina, all physiologically active tissues. Stresses or deficiency of O_2 _(hypoxia) in retinal tissues may cause severe damage, subsequently leading to blinding disease.

The manner in which chronic hypoxia causes cell or tissue damage or cell death has been described in several recent reports documenting that the hypoxia evokes overproduction of ROS, which are a major culprit in cell damage [11] . Moreover, as a physiologically active tissue, the retina requires large quantities of oxygen [11], and fluctuations in oxygen level may alter the entire retinal physiology and lead to failure of homeostasis [11]. Systemic hypoxemia caused by lung or heart disease or a vascular disease in the retina can also cause retinal hypoxia and be a major cause of RGC loss. Recent evidence reveals that chronic hypoxia attenuates the cellular prooxidant-antioxidant balance by accumulation of ROS [63], and this condition has been implicated in progressive neurodegenerative diseases. We believe that chronic hypoxia may be associated with reduced expression and activity of survival molecules and antioxidants such as PRDX6 [9,10,37,42,43,64]. In the present study, we found that RGCs exposed to hypoxia displayed elevated expression of ROS, which was associated with reduced expression of PRDX6 (Figures 3, 4 and 5), and these cells underwent apoptosis (Figure 2). These results are consistent with earlier findings that human pulmonary and coronary artery smooth muscle cells bear higher levels of ROS, and the elevated levels of ROS are a major damaging factor [65]. Moreover, ROS is source of oxidative stress, has gained more and more attention recently because of its role as a cellular signaling for a various molecules released from activated glia or microglia such as glutamate [9], cytokines such as TNF-α and growth factors [9,10]. The production of ROS by these molecules has been associated with activation or deactivation of several transcription factors [8-10,12,43]. We think that elevated levels of inflammatory cytokines in the microenvironment of RGCs are responsible for further elevation of ROS in RGCs that leads RGCs death. Recently, we reported that PRDX6 delivery to RGCs can protect against glutamate or TNF-α mediated cytotoxicity, and that the PRDX6 acts by removing ROS and stabilizing NF-κB activation. Notably, both PRDX6 and NF-κB are producers of ROS. While there is much discussion in the research community about increase or decrease of ROS expression during hypoxia, the present study provides evidence that longer periods of hypoxia generate ROS, and ROS-induced abnormal signaling is a major cause of RGC damage or death (Figures 1, 2 and 3). Our data further support our hypothesis that hypoxia induces ROS; RGCs that over-expressed PRDX6 showed resistance against hypoxic stress, reduced ROS expression, and improved survival. Along with others, we have shown that PRDX6 blocks ROS-mediated pathophysiology that occurs during cataractogenesis, neurological disorders, and diabetic-associated disorders [9,42,43], and plays a pivotal role in maintaining lung cell homeostasis [44,48,66-68]. Moreover, our current study revealed that (i) hypoxia evokes intracellular ROS accumulation, which increases with an increase in time, (ii) ROS elevation is causally related to RGC death, and (iii) PRDX6 can attenuate hypoxia-generated oxidative stress-induced RGC death.

Recently, several reports have shown that hypoxic stress induces ROS production, which, if not quenched, leads to cellular pathophysiology [63,65,69,70]. Using a cell culture system, we found that RGCs exposed to either 1% oxygen (physiological hypoxia) or treated with cobalt chloride, a hypoxia mimetic (chemical hypoxia), display elevated expression of ROS (Figure 2) We observed ~30%-60% RGC death in cells with physiological hypoxia (1% oxygen) and ~30%-70% death with cobalt chloride treatment (Figure 1). These data are consistent with previous studies which observed ~25% RGC death after 12 h of exposure to 5% oxygen [71], ~30% cell death after 24 h of 200 μM CoCl_2 _treatment and found that RGC death following hypoxia was predominantly apoptotic, although both apoptotic and necrotic cell death have been observed [71,72]. Furthermore, in glaucomatous eyes, selective loss of RGCs occurs [73], and these cells are particularly sensitive to systemic hypoxic stress [74] as a result of long-term oxidative damage induced by ROS [75].

Interestingly, our present study found increased expression of PRDX6 for the first 24 h of hypoxia exposure (Figures 4 and 5), but when hypoxia exposure was prolonged, the expression of PRDX6 was reduced, and the reduction was related to cell death. Thus our results demonstrate a novel mechanism of hypoxia regulation of PRDX6, in which concentration and time of exposure of RGCs to hypoxia play pivotal roles in determining the fate of the RGCs, which is dependent upon PRDX6 expression. Our study further demonstrated that elevated levels of ROS in RGCs caused by hypoxia are a major cause of cell death, and that the increase in ROS can be eliminated by over-expression of PRDX6 (Figures 8 and 9). As other Prdxs did not counteract the changes in RGCs, we consider the role of PRDX6 to be pivotal, at least in those cells. Interestingly, we also found that acute hypoxia is beneficial to RGCs, as the acute condition may attenuate the extent of cellular ROS and provide an adaptive control mechanism. If that is the case, RGC death during eye disorders including glaucoma is probably caused by the cumulative effect of hypoxia over time, which produces ROS-driven oxidative damage. Moreover, elevated ROS expression has been observed in rabbit retinal cells during ischemia induced by high IOP [76]. We have reported that lens epithelial cells (LECs) deficient in PRDX6 bear higher levels of ROS, are vulnerable to oxidative stress, and undergo spontaneous apoptosis [37]. Collectively, our results suggest that the reduced expression of PRDX6 in RGCs exposed to hypoxia may be one cause of RGC death. Moreover, ROS-driven oxidative stress has been related to a number of diseases and disorders. In fact, it is possible that most pathology involves oxidative stress, at least to some extent, and this may occur due to suppression of antioxidants such as PRDX6. Our present work has demonstrated that hypoxia suppresses PRDX6 expression, leading in turn to RGC death.

The identification of genes or their products involved in etiology of oxidant-mediated pathology has already led to important insights into the cellular response to stress and mechanisms of oxidant damage. In previous reports, we described the regulation of PRDX6 gene expression by NF-κB [9,10], and the dependence of PRDX6 expression level upon cellular redox state. However, when expression levels of ROS exceed the control of cellular antioxidants, cells die by apoptosis or necrosis. We believe that optimizing the level of ROS by the delivery of PRDX6 should prevent or delay ROS-induced deleterious signaling. Furthermore, the generation of ROS has been associated with the activation and deactivation of the transcriptional protein NF-κB [77,78]. Modulation in the activity of NF-κB in neuronal cells is strongly associated with cellular fate, and NF-κB can have either an antiapoptotic or proapoptotic function [79,80], depending on cell type or cellular microenvironment [29,30,81,82]. Importantly, in RGCs, activation of NF-κB has been found to induce apoptotic signaling, and suppression of its activation significantly enhances the viability of RGCs [30]. Our results also vividly demonstrate that addition of PRDX6 in RGCs attenuates NF-κB activation induced by hypoxia, suggesting that PRDX6 can block the NF-κB-induced death pathway in RGCs. We believe the survival of RGCs is associated with of NF-κB activation in acute hypoxia, since upregulation of PRDX6 would be able to remove ROS, while hyperactivation or inadequate activation of NF-κB in RGCs may be disastrous. Thus, modulation of NF-κB activation should be an important strategy for reducing cellular injury. Overall, it appears that RGC death induced by hypoxia should also be associated with hyper-activation of NF-κB due to higher levels of ROS during glaucoma or other neurological diseases. The activation of NF-κB is seen in various cell types in response to hypoxia. Hypoxia induced activation of NF-κB occurs through IkB activation and its phosphorylation [83,84]. In this study, we found that hypoxia exposure activates NF-κB with its subsequent translocation into nucleus. We also found increased phosphorylation of IkB in RGCs following hypoxia. These findings suggest that NF-κB activation in RGCs under hypoxic conditions involves the activation of the canonical pathway through degradation/phosphorylation of the IkBα. Our data demonstrate that cells over-expressed with PRDX6 protect the RGCs from hypoxia-induced oxidative stress by removing ROS and thereby normalizing NF-κB activation.

## Conclusion

We have found that hypoxia induces ROS-driven RGC death caused by down regulation of PRDX6 in cells under prolonged hypoxia, and ROS expression is causally associated with PRDX6 expression level. Results further revealed that ROS are differentially regulated during hypoxia; however, increased expression of ROS, due to deficiency of PRDX6, indeed reflects pathophysiology of RGCs. Because delivery of PRDX6 may attenuate RGC death by optimizing intracellular ROS and NF-κB activation, PRDX6 should be considered as therapeutic agent for hypoxia-induced disorders. Further detailed research will be needed to elucidate the mechanisms involved in the PRDX6-mediated protection of RGCs.

## Authors' contributions

DPS and NF conception and designing of experiments; RT, LSK, NF, performing experiments and acquisition of experimental data; DPS, NF, RT, LSK, BC, EK, NF, AK, analysis and interpretation of data and writing and revising of manuscripts.
